# Inferior wall ST-elevation myocardial infarction in a patient with a single coronary artery from the right coronary cusp trifurcating into the left anterior descending, left circumflex, and right coronary arteries: a rare coronary anomaly, case report

**DOI:** 10.1093/ehjcr/ytag163

**Published:** 2026-03-06

**Authors:** Al-Naser Al Bahlani, Hammad Ur Rehman, Ayman Al Husini, Mubarak Al Dosari

**Affiliations:** Adult Cardiology Fellow, King Saud Medical City, Saudi Commission for Health Specialties (SCFHS), Riyadh 12742, Saudi Arabia; Interventional Cardiology, King Saud Medical City, Riyadh 12742, Saudi Arabia; Interventional Cardiology, King Saud Medical City, Riyadh 12742, Saudi Arabia; Interventional Cardiology, King Saud Medical City, Riyadh 12742, Saudi Arabia

**Keywords:** Myocardial Infarction, Coronary Vessel Anomalies, Coronary Angiography, Percutaneous Coronary Intervention, Tomography, X-Ray Computed, Case Report

## Abstract

**Background:**

Abnormal origin of a coronary artery is a rare congenital condition that can significantly affect clinical outcomes especially when associated with acute coronary syndromes. Among these, the presence of a single coronary artery trifurcating from the right coronary cusp into all major coronary branches is exceptionally rare and poorly represented in the literature.

**Case summary:**

A 35-year-old man presented with an inferior ST-elevation myocardial infarction. Emergency angiography revealed a single coronary artery arising from the right coronary cusp. The culprit was the right coronary artery. It was effectively treated with intravascular-guided percutaneous coronary intervention, and the remaining coronaries demonstrated normal flow, as shown in subsequent imaging. The patient recovered uneventfully, rehabilitated without complications. He was discharged on guideline directed medical therapy.

**Discussion:**

Although the anomalous coronary anatomy was not the direct cause of infarction, it introduced significant procedural challenges that could have delayed or compromised revascularization. Our case highlights the importance of recognizing and anticipating coronary anomalies in acute settings. Multimodality imaging and anatomical classification systems help provide timely diagnosis, procedural planning, and risk assessment from a long-term perspective.

Learning points

- Recognition of anomalous coronary origins is crucial during primary PCI to avoid catastrophic complications.
- Multimodal imaging, including angiography, CCTA, and IVUS, allows accurate diagnosis and procedural planning.

## Introduction

Anomalous origin of the coronary arteries represents a rare group of congenital anomalies that can have severe clinical consequences.^1^ These anomalies have been reported at a prevalence of 0.3% to 1% of the general population, depending on the imaging modality.^2^ A single coronary artery (SCA), through which all major epicardial coronary arteries arise from a single ostium, is extremely rare. The angiographic prevalence is between 0.024% and 0.3%.^3^ However, there can be a predisposition to ischaemia, arrhythmias, or sudden cardiac death (SCD), particularly when a vessel follows an interarterial or intramural course between the aorta and pulmonary artery.^4^

The purpose of this case report is to present an unusual example of inferior wall ST-elevation myocardial infarction (STEMI) in a patient with a SCA originating from the right coronary cusp. This vessel trifurcated into the left anterior descending artery (LAD), left circumflex artery (LCx), and right coronary artery (RCA), corresponding to the rare Lipton type R-III variant. The report discusses the role of multimodality imaging in clarifying complex coronary anatomy and addressing the procedural challenges of emergent percutaneous coronary intervention (PCI), with particular emphasis on intravascular ultrasound (IVUS) guided PCI. Detailed procedural steps and short-term follow-up are presented. In contrast to most previously reported Lipton type R-III cases, which relied primarily on angiography, IVUS in the present case enabled direct characterization of the mechanism of occlusion and optimization of stent deployment during the intervention.

The clinical significance of coronary anomalies depends more on the origin and course of the anomalous vessel. Exercise-induced ischaemia and SCD in young athletes have been associated with high-risk ‘malignant’ variants, such as an interarterial course.^5^ Conversely, non-malignant variants such as prepulmonic or retroaortic courses generally do not compromise myocardial perfusion but may pose challenges during invasive coronary procedures.^6^ Moreover, reports of PCI in Lipton type R-III subtype cases are minimal, limiting the development of standardized management strategies for STEMI in this rare anatomical setting.^7^

## Summary figure

**Figure:**
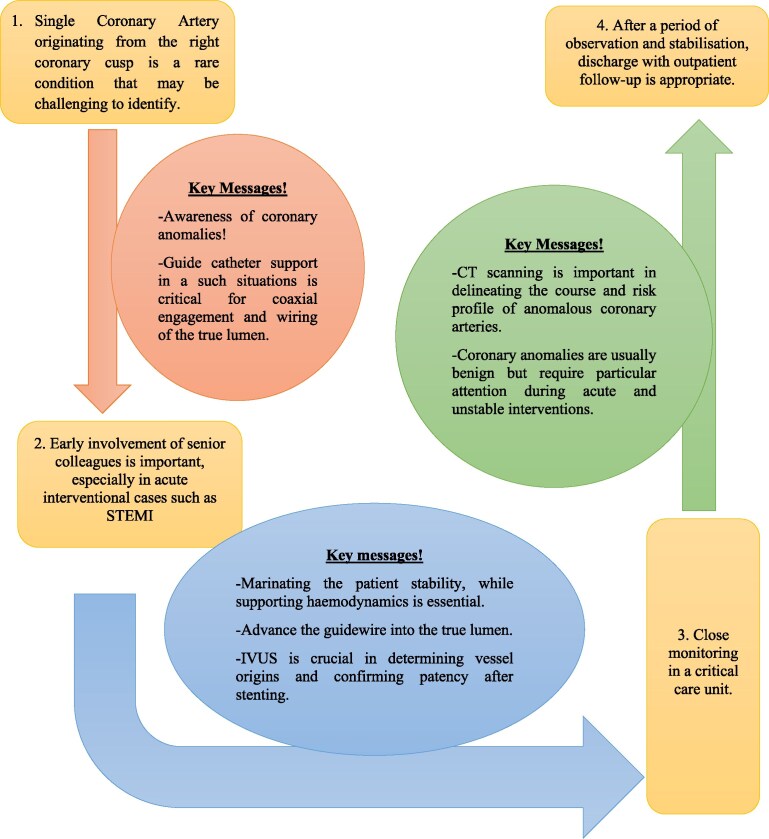


## Case presentation

A 35-year-old male, active smoker, 15 pack-years, without known comorbidity, allergies, or family history of premature coronary artery disease (CAD), reported to the emergency department with acute chest pain. The pain started three hours before the presentation and was characterized as retrosternal and pressure-like. The pain radiated to the left arm and was accompanied by profuse sweating, nausea, and vomiting. No associated syncope, palpitations, or dyspnoea occurred. Upon presentation, he was haemodynamically stable (BP 140/98 mmHg; HR 105 bpm) but in apparent pain. Cardiac and respiratory examinations were unremarkable.

**Figure 1:**
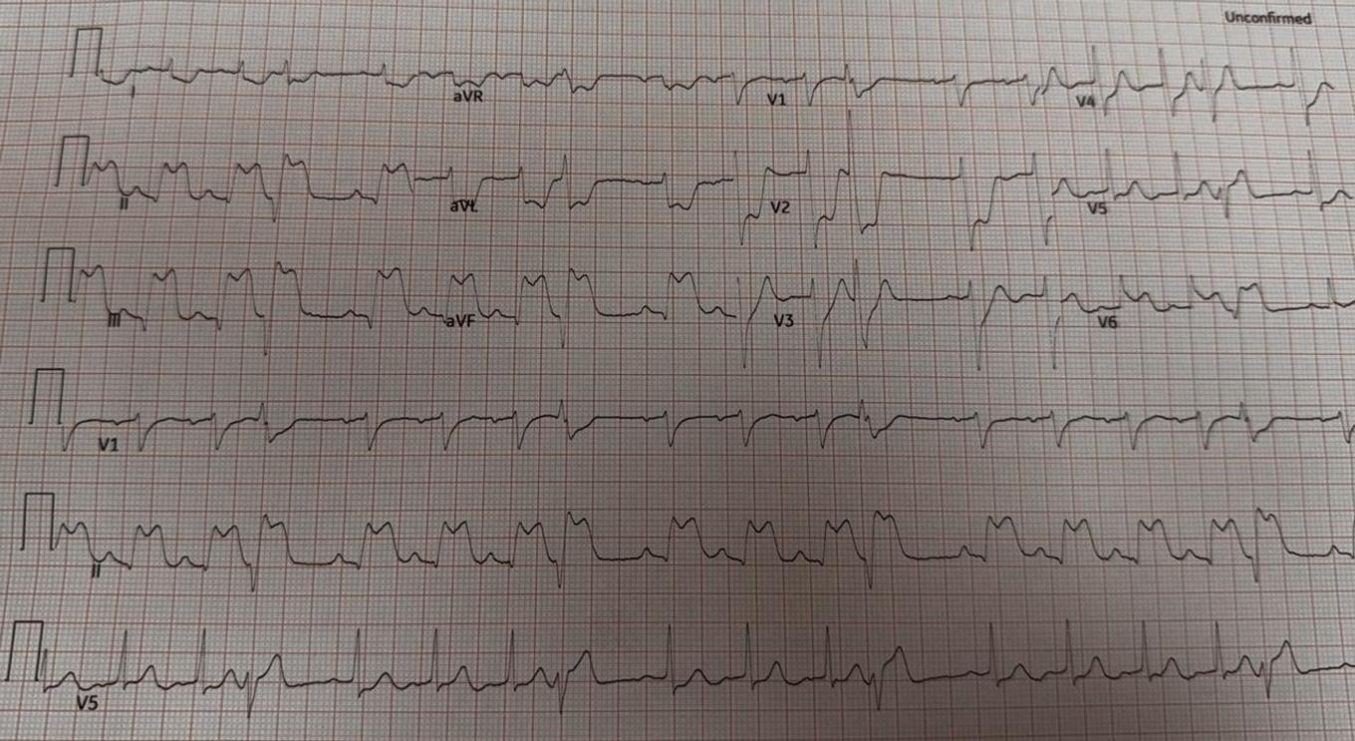
Standard 12-lead electrocardiogram showing ST-segment elevation in leads II, III, aVF and reciprocal changes in leads I and aVL.

The 12-lead ECG revealed significant ST elevation (J point >5 mm) in leads II, III, and aVF, and reciprocal depression in leads I and aVL. V1-V2. Horizontal ST depression was observed (*Figure 1*). V4R ST elevation showed right ventricular involvement, and V7-V9 was a positive indication of posterior extension. These results were compatible with an acute inferior STEMI with right ventricular and posterior involvement. Laboratory results showed significantly increased high-sensitivity troponin I 147.9 ng/L (0–34.2) (ng/L) and slightly increased serum lactate 2.3 mmol/L (0–1.9) (mmol/L). Other routine labs were normal. The chest X-ray had revealed a normal heart size and lungs.

**Figure 2:**
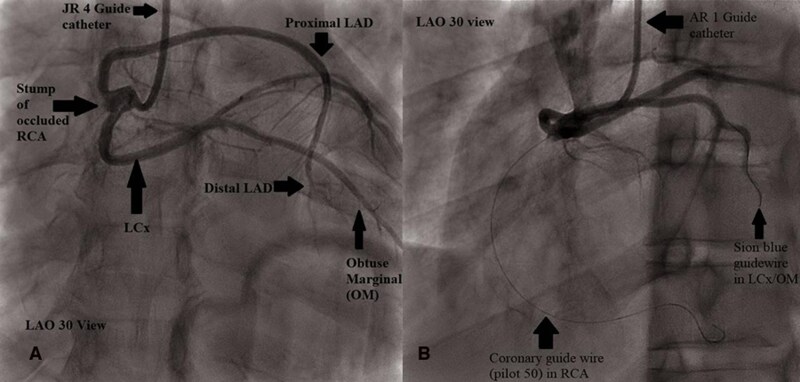
(*A* and *B*): coronary angiography demonstrating single ostium in right coronary cusp giving rise to RCA, LAD, and LCx, with proximal RCA occlusion. Guidewires have been placed into one of the branches (LCx) and occluded vessel, the RCA.

Emergency coronary angiography showed an SCA originating at the right coronary cusp, compatible with Lipton type R-III SCA subtype (*Figure 2*). There was 100% thrombotic occlusion of the proximal right coronary artery with a complete halt of antegrade flow. Given the angiographic appearance, further intraprocedural assessment was undertaken to clarify the underlying cause mechanism of the occlusion, as detailed below.

A Judkins Right 4 (JR4) guiding catheter was initially used; however, engagement and guide support were felt to be suboptimal, therefore, an Amplatz Right 1 (AR1) guiding catheter was selected to provide more support. The lesion was crossed with a Pilot 50 coronary guidewire.

**Figure 3:**
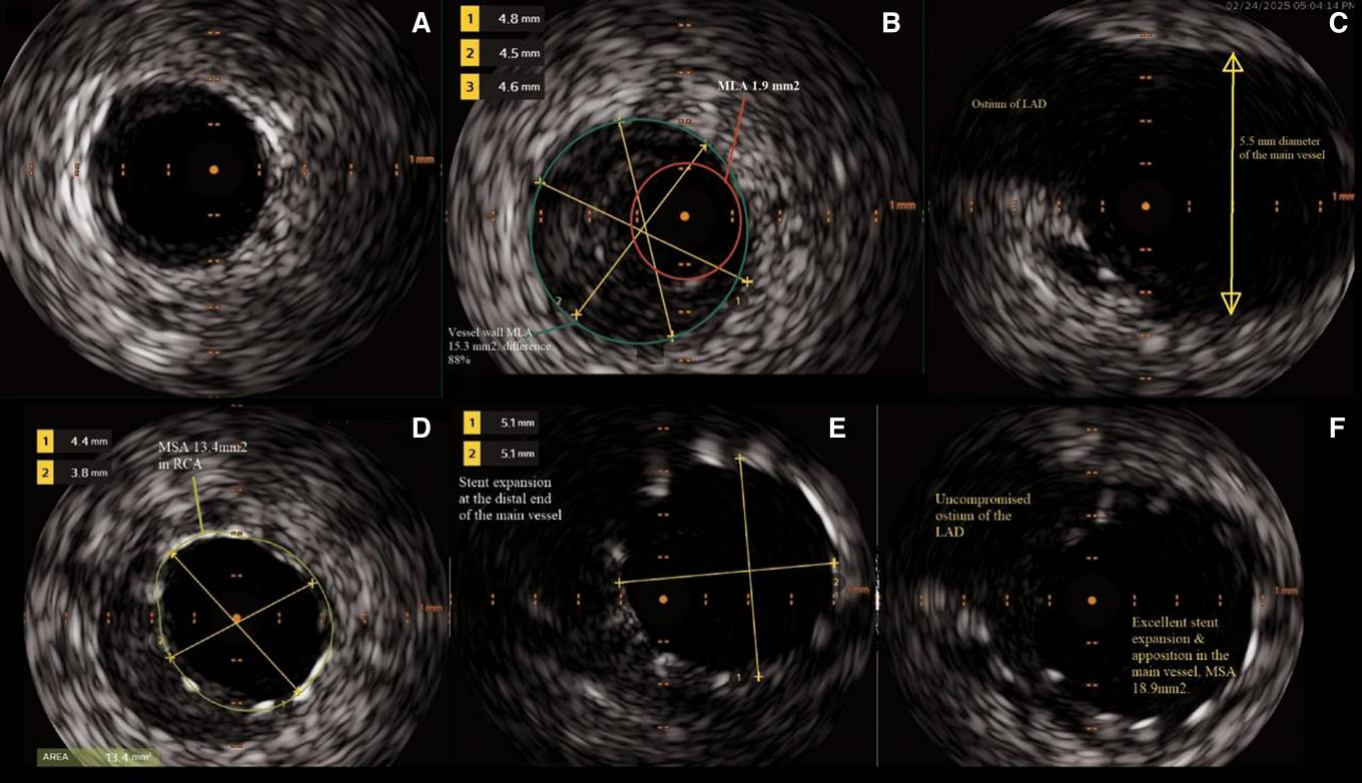
IVUS images prestenting (panels *A*, *B*, *C*) and post stenting (panels *C*, *D*, *E*); (*A*) shows the distal RCA which has healthy arterial layers and no visible plaque. (*B*) Depicts plaque rupture with heavy plaque burden at the RCA ostium (plaque burden 88%) with a reference vessel diameter of 4.5 mm, precluding safe proximal stent landing at this site because of severe disease. (*C*) Proximal main vessel is healthy, and ostium of LAD is visible at 11 o’clock position. (*D*) Stent expansion and apposition in RCA is satisfactory while plaque is visible behind the stent. (*E*) Distal end of the main vessel is showing residual plaque and stent expansion of 5 mm. (*F*) The LAD ostium is uncompromised and the main vessel MSA is 18.9 mm^2^, with excellent stent apposition and expansion.

**Figure 4:**
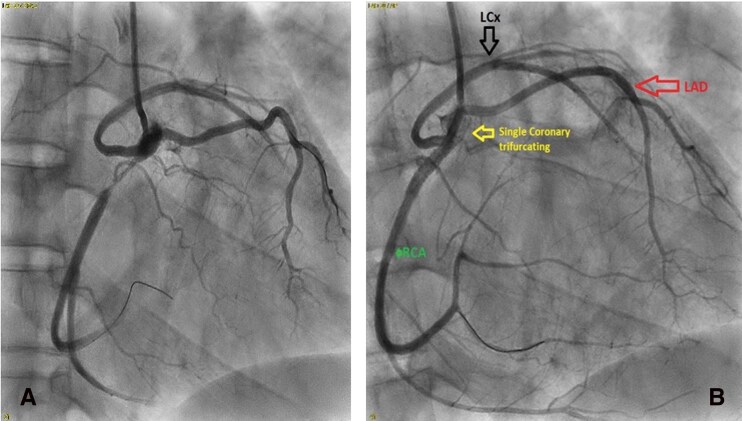
Post-intervention angiography with restored flow after balloon inflation (*A*) and with drug-eluting stent in proximal RCA and restored TIMI III flow (*B*) with no compromise of flow in other vessels.

IVUS done after recanalizing the artery demonstrated a severely atherosclerotic vessel with plaque burden approximately 90% and a minimal luminal area (MLA) of 1.9 mm^2^, followed by healthy vessel a few millimetres distal to the lesion. The culprit lesion was identified as plaque rupture with overlying thrombus at the ostium of the RCA (*Figure 3*). Based on these findings, a decision was made to stent across the lesion while covering the main vessel. A drug-eluting stent (DES) (4.0 × 28 mm SYNERGY) was deployed followed by proximal optimization with 5 × 10 mm non-compliant (NC) balloon and further post dilatation of the RCA using 4 × 15 mm NC balloon. Final IVUS demonstrated a well-apposed stent, with a minimal stent area of 18.9 mm^2^ in the main vessel and 13.4 mm^2^ in the RCA, without IVUS or angiographic compromise of the ostia of the LAD and LCX (*Figure 4*). A total of 140 mL of iso-osmolar contrast (iodixanol) was used which was within acceptable limits for a complex primary PCI.

**Figure 5:**
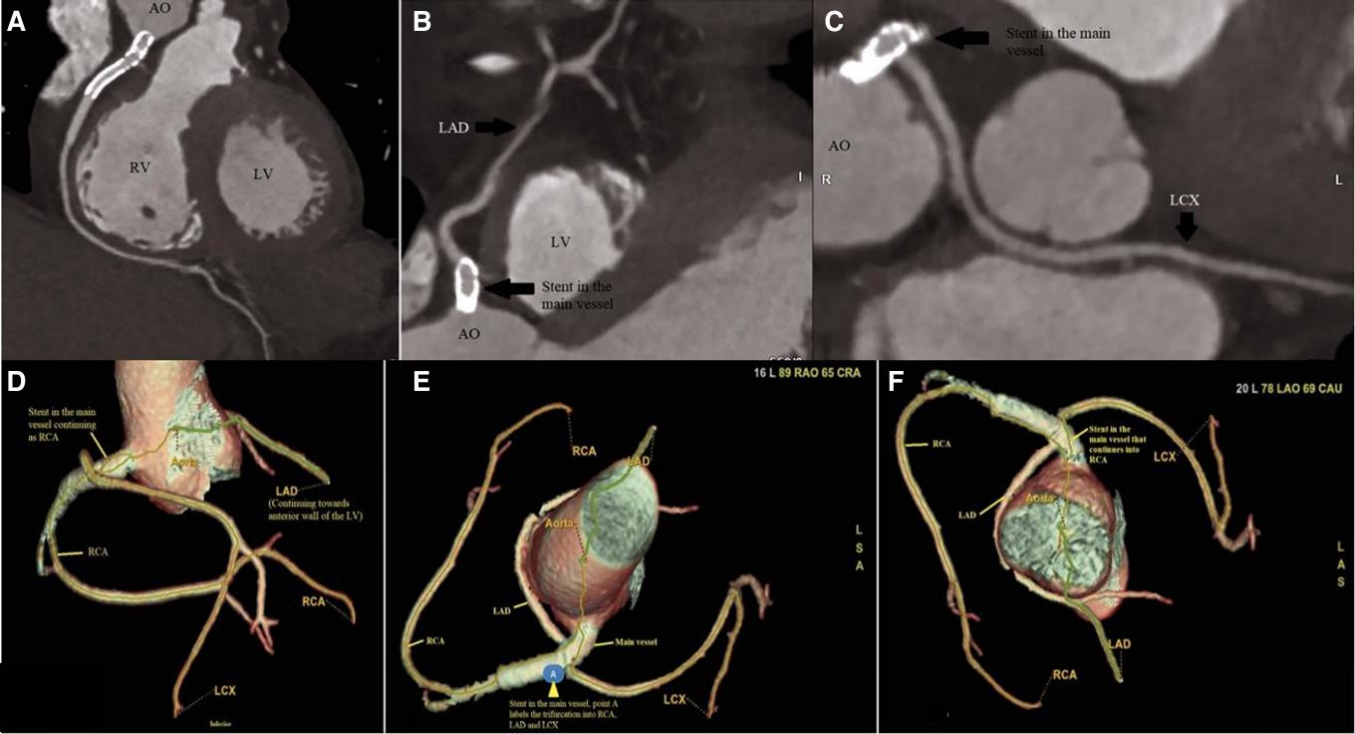
CT reconstruction, including three-dimensional views, of coronary arteries arising from the right coronary artery. (*A*) RCA showing patent stent in the ostio-proximal portion of the artery followed by normal course of a dominant RCA. (*B*) LCx arises from the right coronary artery and supplies posterolateral wall and has a benign course. (*C*) The LAD arising from the RCA at the ostial portion of which the deployed stent is visible and the vessel supplies anterior wall and anterior septum of the LV. (*D*) Right anterior oblique view demonstrating the stented main vessel, LAD arising from the right coronary artery and coursing anteriorly towards the anterior wall of the left ventricle. (*E*) Superior view of the aorta showing the single coronary ostium at the right coronary cusp, with the stented main vessel visible; point A marks the site of trifurcation into the RCA, LAD, and LCx. (*F*) Additional superior view of the aorta further delineating the course of the coronary arteries relative to the great vessels.

Coronary anatomy was assessed using coronary computed tomography angiography (CCTA) following PCI. Imaging revealed right coronary dominance and a lack of the left main coronary artery (LM). The LAD was observed to arise out of the RCA with a benign prepulmonic route and low non-calcified plaque burden, whereas the LCx source was also from the RCA with a benign retroaortic course and minimal stenosis. The RCA stent was patent with a minimal distal plaque burden as shown in *Figure 5*.

After PCI, the patient was observed in the coronary care unit. The chest pain was resolved in a few hours, and the ECG showed resolution of ST elevation. Transthoracic echocardiography revealed normal left ventricular function (EF ∼55%), mild inferior wall hypokinesia, normal right ventricular function, and no significant valvular pathology. He was initiated on dual antiplatelet therapy, high-intensity statin, beta-blocker, and ACE inhibitor, and he received counselling on smoking, diet, and activity. He was stable at the time he was discharged on the fifth day. Two months later, he was asymptomatic with intact ventricular function, participated in cardiac rehabilitation, and had no complications. He was scheduled for regular follow up, with either coronary CT angiography or myocardial perfusion imaging planned as part of ongoing surveillance.

## Discussion

There are limited reports describing acute coronary syndrome (ACS) in patients with SCA.^8^ The Lipton R-III variant, in which the LAD, LCx, and RCA originate from a single ostium in the right coronary cusp, represents one of the least frequently reported subtypes in the Lipton classification.^1^ Previously published Lipton R-III cases presenting with acute coronary syndromes are few and are summarized in *Table 1*, underscoring the rarity of this anatomical pattern in the setting of acute myocardial infarction.

**Table 1:** Previously reported lipton R-III single coronary artery cases

Author (Year)	Presentation	Culprit	Mechanism	Management	Outcome
Varol S *et al*. (2015)	Lateral STEMI	LCx	Plaque rupture	PCI to LCx with DES	Discharged, good outcome
Shizukuda Y *et al*. (2016)	ACS, high blood pressure	None (Moderate CAD)	Atherosclerosis	Medical treatment	Discharged, no more episodes of pain.
Kehran *et al*. (2017)	Stable angina	None	No occlusion found	Benign Course of LAD and LCx	No data
Guria RT *et al*. (2017)	Transient Anterior STEMI	LAD	LAD had intramyocardial course	Medical therapy	Patient was advised CABG, but refused on annual follow up
Lalani K *et al*. (2022)	IWMI	RCA	Plaque rupture	Angioplasty	Discharged, good outcome
Popa RM *et al*. (2023)	ACS	LCx	Atherosclerosis	PCI to LCx with DES	Discharged, good outcome
Gupta A *et al*. (2024)	Stable angina	RCA CTO	Atherosclerosis	PCI to RCA with DES	Discharged, good outcome
**Present case**	IWMI	RCA	Plaque rupture	IVUS-PCI to RCA with DES	Discharged, full recovery, on regular follow up

In the present case, the culprit lesion was a thrombotic occlusion of the proximal RCA, a finding that has been described in other reported cases of acute coronary syndromes.^9^ Although the anomalous LAD and LCx followed benign courses, this does not preclude the risk of acute coronary occlusion or its significant clinical consequences.^10^

PCI in SCA requires heightened procedural caution because a single coronary ostium supplies the entire myocardium. Catheter selection is critical, as adequate guide support must be balanced against the risk of ostial trauma or dissection, which may result in global myocardial ischaemia. Anomalous vessel origin and angulation can further limit coaxial engagement and device delivery, potentially prolonging procedural time and increasing contrast exposure, particularly in complex interventions. These considerations highlight the importance of meticulous technique and careful procedural planning in patients with SCA anatomy.^11^

In contrast to most previously reported R-III cases, which relied primarily on angiographic assessment, the present case incorporated IVUS for lesion characterization and procedural guidance. IVUS demonstrated an atheromatous plaque with overlying thrombus in the proximal RCA, confirming plaque rupture as the mechanism of acute occlusion, and excluded alternative causes such as spontaneous coronary artery dissection. Furthermore, IVUS guided appropriate stent sizing and proximal optimization to ensure adequate expansion and apposition, while preserving flow to the anomalous LAD and LCx branches.^12,13^

Given the rarity of SCA and the potential consequences of recurrent ischaemia, long-term management should emphasize on aggressive secondary prevention and close clinical follow-up. Multimodal imaging plays an important role in post-procedural assessment and anatomical definition, and consideration may be given to non-invasive stress testing and CCTA for surveillance in selected patients, particularly those with complex coronary anatomy or recurrent symptoms.^1,14^

Post-procedural CCTA confirmed the benign prepulmonic course of the LAD and the retroaortic course of the LCx, as well as stent patency. Such benign courses are generally incidental findings and are associated with favourable long-term outcomes. In contrast, malignant coronary courses, including interarterial or intramural trajectories, are associated with an increased risk of myocardial infarction, arrhythmias, and exercise-related sudden cardiac death, particularly in younger individuals.^14^ However, much of the evidence supporting the prognostic implications of malignant coronary courses is derived from autopsy studies, athlete registries, and small observational series, which may not accurately reflect long-term risk across broader populations. Autopsy data have nevertheless demonstrated that anomalous coronary arteries may account for a substantial proportion of sudden cardiac death in young athletes, reported in up to 17% of cases in a large series.^15^

Overall, the prognosis of patients with Lipton R-III anatomy remains incompletely defined owing to the small number of reported cases treated with PCI, heterogeneity in anatomical characterization, and reliance on case-based evidence.

## Patient perspective

The patient had a quick recovery without complications.

## Conclusion

This case underscores the importance of recognizing and understanding rare SCA anatomy in patients presenting with STEMI. Although the infarction in this case was caused by plaque rupture, the anomalous coronary anatomy introduced additional procedural challenges during emergent intervention. Early anatomical identification, supported by multimodality imaging, is essential to guide catheter-based strategies and minimize procedural risk. This report adds to the limited literature on Lipton type R-III anatomy and highlights the need for a high index of suspicion for atypical coronary origins in acute settings.

## Data Availability

Images and video materials supporting the findings of this case report, including intravascular ultrasound clips, are available via the following secure link: [10.6084/m9.figshare.30911237]. Additional data are available from the corresponding author upon reasonable request.

## Lead author biography

Dr. Al-Naser Al Bahlani is an adult cardiology trainee at King Saud Medical City/Riyadh. His clinical and research interests include coronary artery anomalies, coronary intervention, heart failure, arrhythmia, and syndromic cardiac conditions. He is particularly interested in the role of multimodality imaging in guiding diagnosis and management of complex cardiovascular disease. Dr. Al-Naser aims to pursue a career in academic and interventional cardiology, integrating clinical practice with research and teaching.

## Notes

**Consent:** Written informed consent for publication of this case report and accompanying images was obtained from the patient in accordance with COPE guidelines.
